# Supplementary material for: Domain sliding of two Staphylococcus aureus N-acetylglucosaminidases enables their substrate-binding prior to its catalysis
Source: Commun Biol. 2020 Apr 20;3:178. doi: 10.1038/s42003-020-0911-7 (PMC7170848; doi:10.1038/s42003-020-0911-7)
Supplement: Supplementary file 1 — Supplementary Information [file 42003_2020_911_MOESM1_ESM.pdf]

Supplementary information

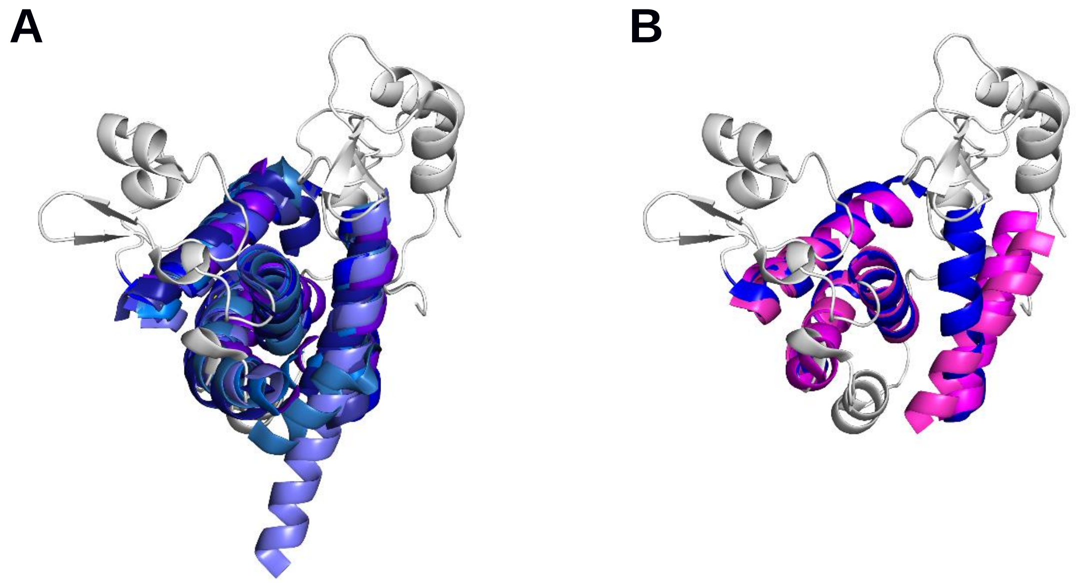

Supplementary Figure 1: The comparison of the positioning of the core helices in the GH73 family of enzymes. (A) All the core helices (in different shades of blue) of all the other GH73 structures, namely Acp (5WQW), Auto (3FI7), StFlgJ (5DN4), SpFlgJ (3VWO), ScaH (5T1Q), LytB (4Q2W) and TM0633 (4QDN), are aligned to the core helices of AtlE (the rest of the structure is in white). (B) The core helices of SagB and AtlA-gl (in magenta shades) are superimposed to the core helices of AtlE (in blue). (The superimposition aligned the helices in the L-domain, whereas the SagB and AtlA-gl helices of the L-domain are shifted relative to the AtlE helices of the R-domain.) The positioning of the core helices of all the other structures is very similar, while the positioning of the equivalent helices is SagB and AtlA-gl is visibly different.

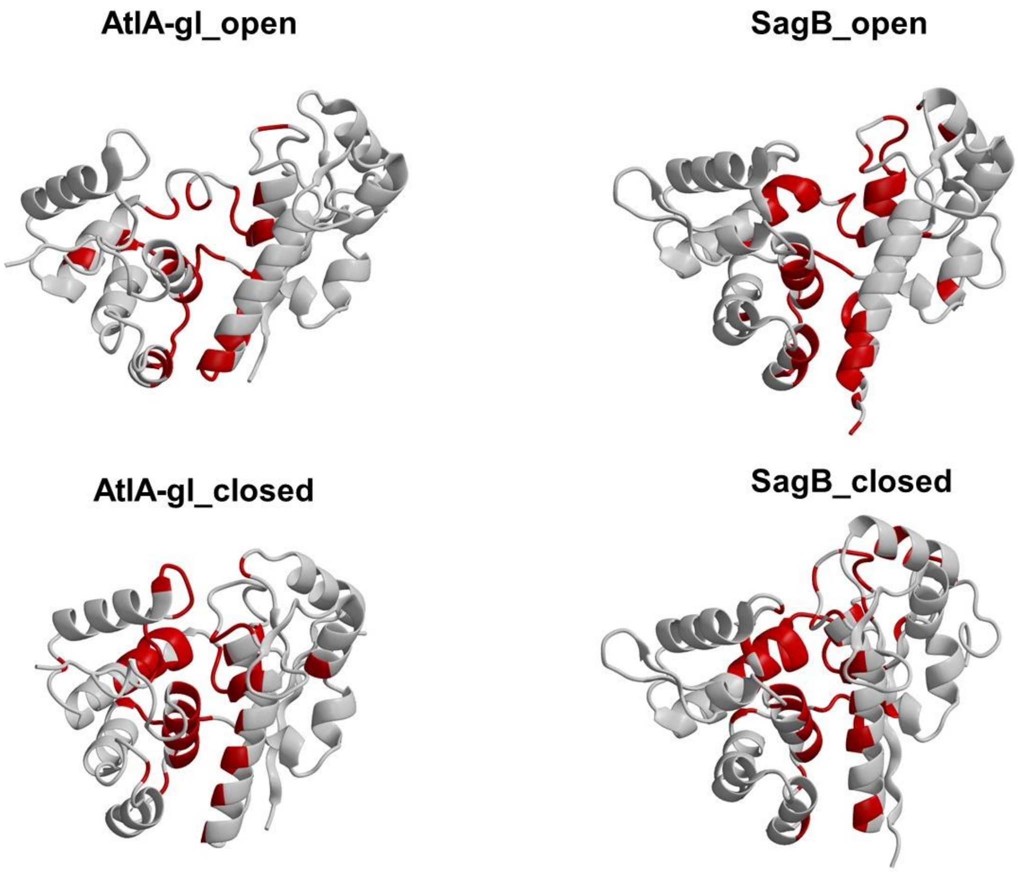

Supplementary Figure 2: Visualization of specific inter-residue contacts revealed their localization along the two domain interfaces of AtlA-gl and SagB structures. In front, in the open forms, the specific contacts are located at the bottom of the R-domain long helices, whereas in the closed forms, they were positioned higher at interactions with the central helices, where the termini catalytic Glu resides are positioned. At the back, during the transition, the substrate binding region residues changed their contact partners.

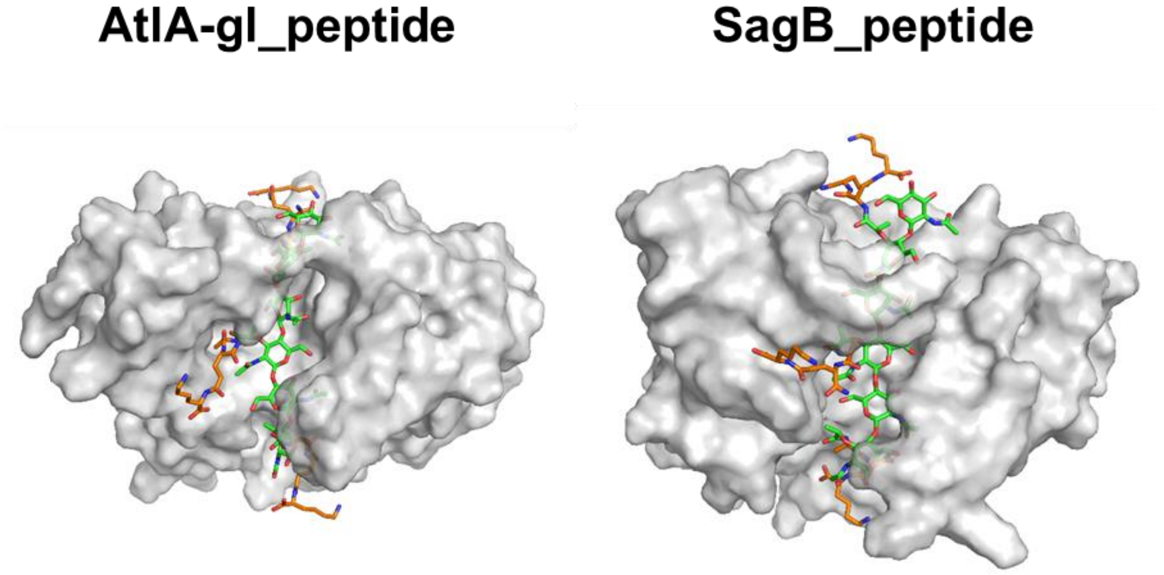

Supplementary Figure 3: 3D model of the substrate, that consists of glycan strand (in green) and added L-Ala-D-iso-Gln-L-Lys part of the peptide stem (in orange) bound to the closed models of the structures. The peptide stems were modelled onto the glycan strand and subsequently minimized. It is evident that the presence of the peptide stems allows the binding of the substrate as well as it does not hinder the closing of the enzymes.

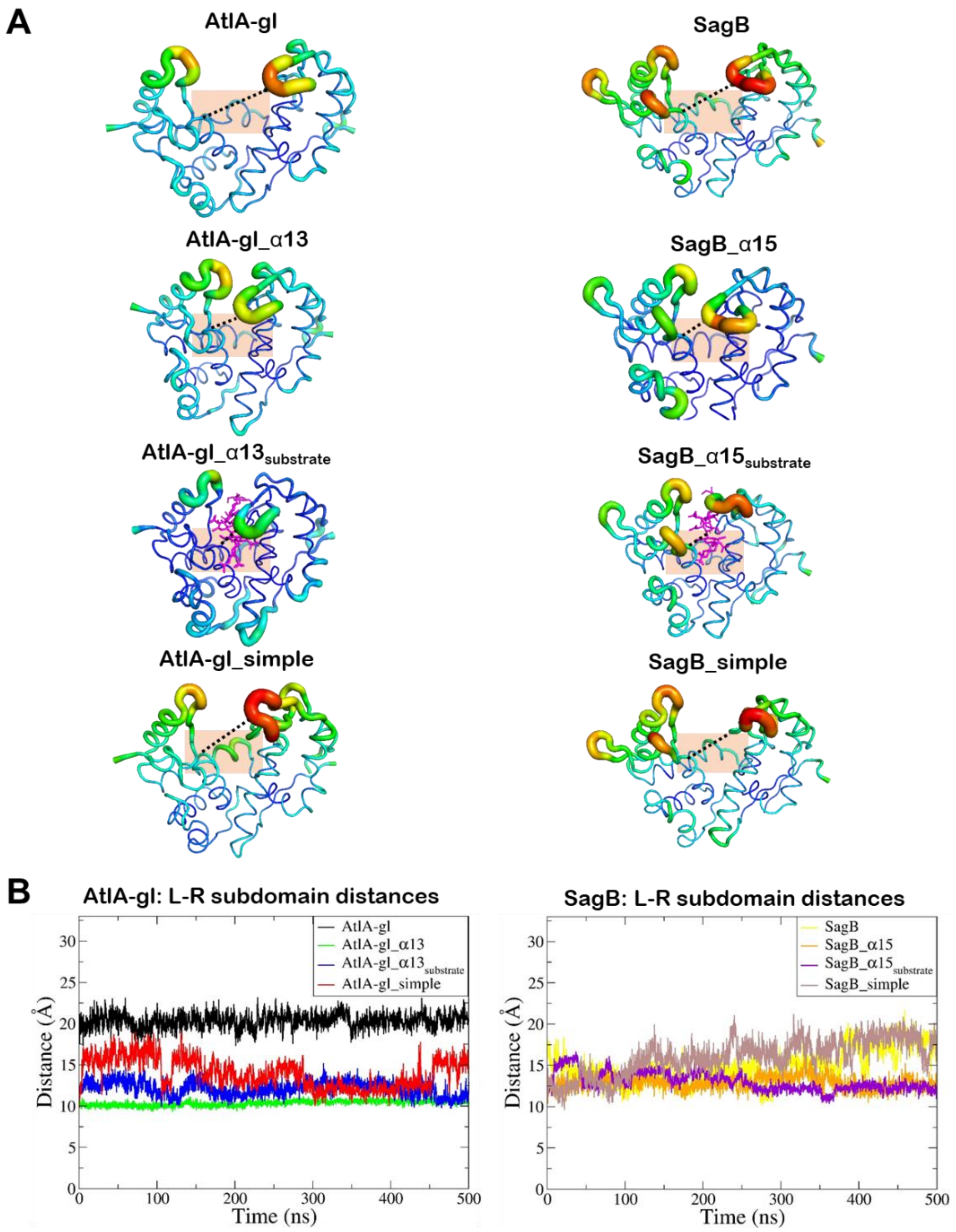

Supplementary Figure 4: Molecular dynamics (MD) simulations of the crystal structures, generated closed structures with and without elongated helices, and closed structures with elongated helices including the (NAG-NAM)$_3$ substrate for the AtlA and SagB enzymes. (A) Flexibility of distinct AtlA-gl and SagB models extracted from the MD trajectories. AtlA-gl and SagB showing closed structures with and without elongated helices. Representative conformations of each simulated case with their monitored L-R domain distance marked. The orange box designates the region of differences among the helices. (B) Time-dependent graphs displaying the measured L-R domain distances for all simulated AtlA-gl and SagB systems.

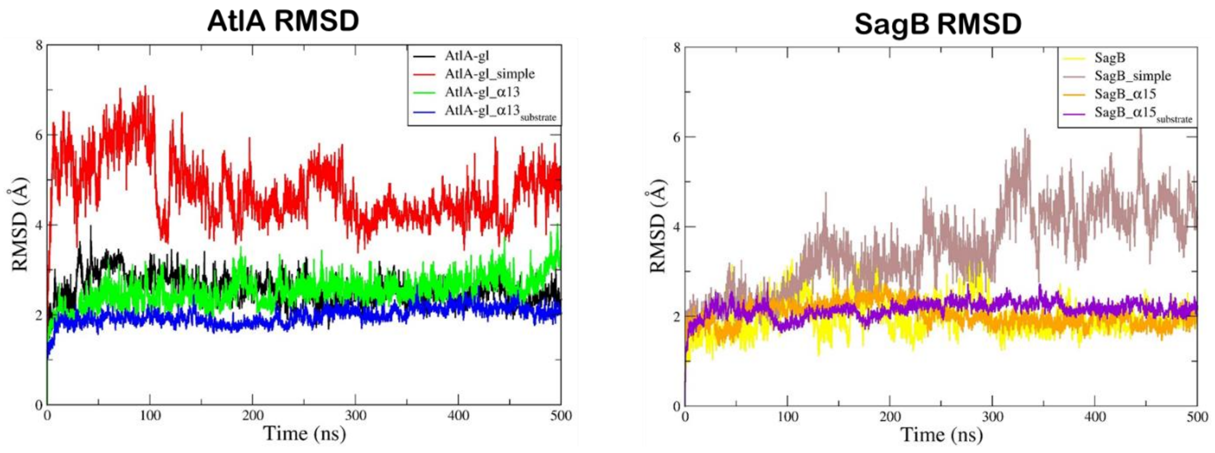

Supplementary Figure 5: RMSD plots for C-α atoms of all conducted simulations of AtlA and SagB systems.

Supplementary Table 1: RMSD C-α atoms values) and average distances between the L- and D- lobes for all conducted simulations of AtlA and SagB systems.

**System**	**RMSD (Å)**	**Distance (Å)**
AtlA-gl	2.56 ± 0.32	20.3 ± 0.91
AtlA-gl_α13	2.53 ± 0.32	12.2 ± 0.87
AtlA-gl_α13$_{substrate}$	1.98 ± 0.19	10.4 ± 0.32
AtlA-gl_simple	4.76 ± 0.71	14.0 ± 1.8
SagB	1.99 ± 0.41	14.9 ± 2.01
SagB_α15	2.03 ± 0.25	12.9 ± 0.78
SagB_α15$_{substrate}$	2.15 ± 0.18	13.0 ± 1.14
SagB-simple	3.52 ± 0.94	16.0 ± 2.2

Supplementary Table 2: Primers used for cloning in the study.

protein	sense	antisense
SagB-wt	TACTTCCAATCCAATGCCGCAGCAttcaaacatgttaaatccga	TTATCCACTTCCAATGTTAttacttattcaaatgtttac
AtlA-gl-wt	TACTTCCAATCCAATGCCGCAGCAgaattaattaagtataatcaa	TTATCCACTTCCAATGTTAtttatattgtgggatgtcg
AtlA-gl-E116A	tctcacatgccctattagcaacaggtaacggtacttc	gaagtaccgttacctgttgctaatagggcatgtgaga
AtlA-gl-Y214A	tcctgcacatccaggaacacaccaagctgctacagatgtag	ctacatctgtagcagcttggtgtgttcctggatgtgcagga
AtlA-gl-Y214F	cacatccaggaacacaccaatttgctacagatgtagatt	aatctacatctgtagcaaattggtgtgttcctggatgtg
AtlA-gl-T216A	ggaacacaccaatatgctgcagatgtagattgggcta	tagcccaatctacatctgcagcatattggtgtgttcc
AtlA-gl-D217A	acacaccaatatgctacagctgtagattgggctaacatc	gatgttagcccaatctacagctgtagcatattggtgtgt
AtlA-gl-D217N	gaacacaccaatatgctacaaatgtagattgggctaacatc	gatgttagcccaatctacatttgtagcatattggtgtgttc
AtlA-gl-Y191A	gtggtgctaaattcatcggcaactcagctgtaaaagctggtcaaa	tttgaccagcttttacagctgagttgccgatgaatttagcaccac
AtlE-Y201A	ttggtggtgcaaagttcattcgtaatgaagcttttgaaaacaatcaactgaatttatatca	tgatataaattcagttgattgttttcaaaagcttcattacgaatgaactttgcaccaccaa
