# Supplementary material for: Domain sliding of two Staphylococcus aureus N-acetylglucosaminidases enables their substrate-binding prior to its catalysis
Source: Commun Biol. 2020 Apr 20;3:178. doi: 10.1038/s42003-020-0911-7 (PMC7170848; doi:10.1038/s42003-020-0911-7)
Supplement: Supplementary file 2 — Description of Additional Supplementary Files [file 42003_2020_911_MOESM2_ESM.pdf]

Description of Additional Supplementary Files

File Name: **Supplementary Movie 1**
Description: The transition of AtlA-gl from the open to the closed conformation. Active glutamate (E116) is depicted in red and the substrate binding YATD (Y214-D217) region in blue.

File Name: **Supplementary Movie 2**
Description: The transition of SagB from the open to the closed conformation. Active glutamate (E121) is depicted in red and the substrate binding YATD (Y209-D212) region in blue.

File Name: Supplementary Data 1
Description: Source data underlying the plots shown in Figure 4
